# eHealth and mHealth initiatives in Bangladesh: A scoping study

**DOI:** 10.1186/1472-6963-14-260

**Published:** 2014-06-16

**Authors:** Tanvir Ahmed, Henry Lucas, Azfar Sadun Khan, Rubana Islam, Abbas Bhuiya, Mohammad Iqbal

**Affiliations:** 1 Centre for Equity and Heath Systems, International Center for Diarrhoeal Disease Research, Bangladesh (ICDDR,B)68, ShaheedTajuddin Ahmed Sarani, Mohakhali, Dhaka 1212, Bangladesh; 2 Institute of Development Studies, University of Sussex (UoS), Library Road, Brighton BN1 9RE, UK

**Keywords:** eHealth and mHealth in Bangladesh, Electronic health and Bangladesh, Mobile health and Bangladesh, Telemedicine in Bangladesh

## Abstract

**Background:**

The health system of Bangladesh is haunted by challenges of accessibility and affordability. Despite impressive gains in many health indicators, recent evidence has raised concerns regarding the utilization, quality and equity of healthcare. In the context of new and unfamiliar public health challenges including high population density and rapid urbanization, eHealth and mHealth are being promoted as a route to cost-effective, equitable and quality healthcare in Bangladesh. The aim of this paper is to highlight such initiatives and understand their true potential.

**Methods:**

This scoping study applies a combination of research tools to explore 26 eHealth and mHealth initiatives in Bangladesh. A screening matrix was developed by modifying the framework of Arksey & O’Malley, further complemented by case study and SWOT analysis to identify common traits among the selected interventions. The WHO health system building blocks approach was then used for thematic analysis of these traits.

**Results:**

Findings suggest that most eHealth and mHealth initiatives have proliferated within the private sector, using mobile phones. The most common initiatives include tele-consultation, prescription and referral. While a minority of projects have a monitoring and evaluation framework, less than a quarter have undertaken evaluation. Most of the initiatives use a health management information system (HMIS) to monitor implementation. However, these do not provide for effective sharing of information and interconnectedness among the various actors. There are extremely few individuals with eHealth training in Bangladesh and there is a strong demand for capacity building and experience sharing, especially for implementation and policy making. There is also a lack of research evidence on how to design interventions to meet the needs of the population and on potential benefits.

**Conclusion:**

This study concludes that Bangladesh needs considerable preparation and planning to sustain eHealth and mHealth initiatives successfully. Additional formative and operational research is essential to explore the true potential of the technology. Frameworks for regulation in regards to eHealth governance should be the aim of future research on the integration of eHealth and mHealth into the Bangladesh health system.

## Background

Access to quality health services and associated costs are a threat to Bangladesh’s current momentum for universal health coverage (UHC). The existing health system is largely (>60%) dependent on out-of-pocket payments [1]. Among many health system concerns, a serious lack and unequal distribution of qualified health human resources (HHR) [2] is a harsh reality. Only 25% of the HHR is working for the rural population which accounts for 70% of the total population [3]. Furthermore, high population density and rapid urbanization is resulting in new and unfamiliar public health challenges [4]. Despite impressive gains in a number of health indicators, recent evidence suggests limited and inequitable access and utilization of quality health services, issues that are central to any effective health system [5].

Given the assumption that a combination of tools can better equip health care providers, enhance the quality of care and reduce existing disparities in health [6,7], electronic health (eHealth) and mobile health (mHealth) have rightly gained considerable attention as a potential tool for healthcare delivery. eHealth and mHealth have been defined in many ways that essentially confer more or less similar attributes [8-10]. eHealth is an umbrella that includes a spectrum of technologies including computers, telephony and wireless communications to provide access to health care providers, care management and education [11]. mHealth is essentially a subset that delivers such services via mobile phones [12,13]. In brief, eHealth and mHealth facilitate provision of healthcare through information and communication technology. Globally, eHealth is steadily becoming a popular platform for healthcare delivery [3] and Bangladesh is no exception. A number of initiatives have already been implemented since the late 90’s. These have mainly focused on mobile phones, especially important amongst the rural and underserved communities for their potential to overcome geographical boundaries. In 2011, WHO reported Bangladesh as one of the 15 countries using mHealth to raise health awareness [14]. As for effectiveness, there is still insufficient evidence regarding the role of eHealth and mHealth in improving access to and/or affordability of preventive, curative or rehabilitative services [15].

Bangladesh is currently in the process of adopting a framework for eHealth and mHealth, based on a decade of experience [16]. In seeking to integrate eHealth and mHealth in the current health system, the main challenge is to address issues related to implementation, i.e. the nature of the services that need to be provided, financial viability of the initiatives and staffing required [17,18]. We have attempted to identify existing eHealth and mHealth initiatives prior to the integration process in order to document the features that would benefit from government action. The foremost research questions in this context are: who are the subsisting eHealth and mHealth actors in Bangladesh; and what spectrum of services are being covered? Considering the current policy interest, it is a prime time to explore the actual and potential contribution of existing eHealth initiatives and the contexts within which they have been implemented. The aim of this paper is to produce an inventory of such initiatives and discuss the challenges for integration of eHealth in Bangladesh. This can not only inform the development of the eHealth framework discussed above but can also act as a prelude to future systematic reviews in this field.

## Methods

**Figure 1:**
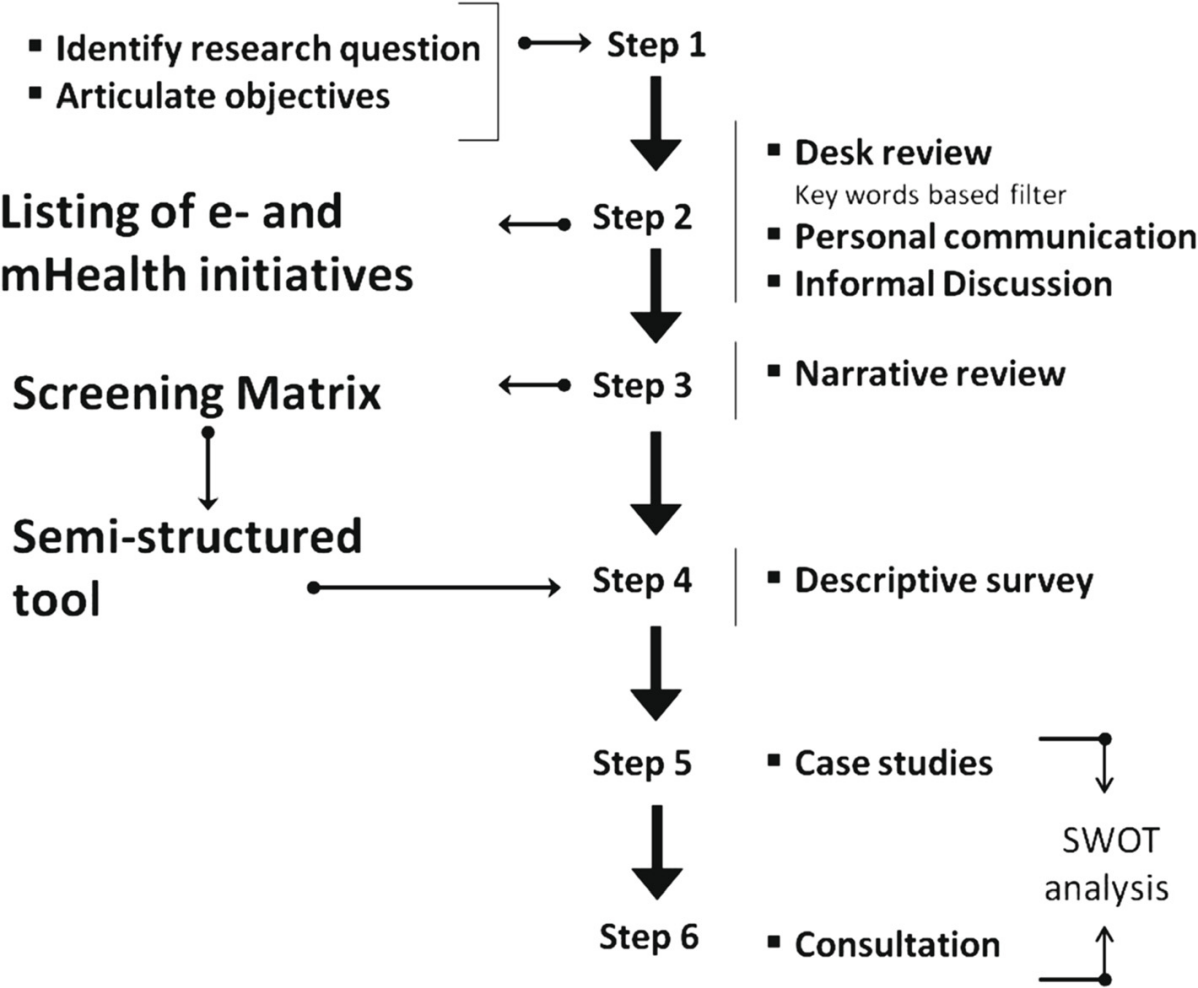
The study design.

Conducted over the period January to March 2012, the study set out to rapidly map extant information on eHealth and mHealth in Bangladesh. A scoping design was chosen as it allowed assimilation of relevant but un-standardized and multiform sources of information, i.e. peer reviewed articles, grey literature (government reports, reports of consultants etc.), conference presentations and proceedings, informal discussions and relevant websites. Such a flexible strategy requires careful design to ensure rigor. The present study was therefore designed around a methodological framework developed by Arksey and O’Malley [19] with some pertinent adjustments. For example, Arksey and O’Malley considered studies intended only for literature review, but for the purpose of this study a short descriptive survey on listed interventions was added to aid in data authenticity. It is very important that in a flexible design each tool should be crafted in a logical sequence using a process of triangulation [20]. Figure 1 shows the steps of the design.

The study began by identifying research question and specific objectives. This was followed by a web search of relevant documents on eHealth and mHealth initiatives in Bangladesh. The search was performed in Google & Google Scholar using keywords e.g. ‘eHealth and mHealth in Bangladesh,’ ‘electronic health Bangladesh,’ ‘mobile health Bangladesh’ and ‘telemedicine in Bangladesh’. Documents dating from 1990 to the present were reviewed. This was used to draft a preliminary list of eHealth and mHealth intervention/programs, which was later revised through personal communication and informal discussions. All the initiatives identified through the desk review were analyzed to prepare a matrix (step 4 of the Arksey & O’Malley framework [19,21]) to outline common traits and themes, i.e. name and type of the institution, technology used, status of the initiative, focus (vision, mission, objectives), target population, financial information, service provision etc. A semi-structured questionnaire was designed using the above matrix. The themes used for data compilation and analysis were organization profile, program overview, financial profile, human resource, services, MNE framework, sustainability plan and future direction. Table 1 shows the breakdown of each themes.

**Table 1:** Themes for data compilation and analysis used in the study

**Theme**	**Breakdown**
**Organization profile**	Managed by (Govt., Private, NGO), Entity (for profit; commercial/non-commercial, not for profit), Priority (health over telecom, telecom over health), Donors & Partners (banks, research organizations, media, telecom etc.)
**Program overview**	Project Status (Planned, ongoing, completed), Main Component (eHealth/mHealth), Medium (SMS/voice call/computer/other), Technological platform (software, hardware), Target Population (e.g. rural villagers, age groups etc.), Measure of Utilization (# of people registered, # of people using services)
**Financial**	Startup Cost, Operational Cost, Total Revenue
**Human resource**	Type and number of staff and training
**Services**	Disease management (disease covered, mode of provision, provider, cost), Health awareness (topics covered, mode of provision, provider type, cost), Diagnostic/Imaging (tests covered, mode of provision, provider, cost)
**MNE Framework**	Indicators (input/process/output/outcome/impact), Project Evaluation (if done - main Findings and their uptake)
**Sustainability plan**	Strategy (plans created for Present undertakings, upcoming deals or work in progress arriving shortly, that drives the institution forward in the defined sector), Capacity (resources and infrastructure support required/available to sustain the strategy and future direction in the defined sector)
**Future direction**	Major challenges (if no sustainability plan is evident, try to find out what were the major challenges that hampered sustainability)

The findings were presented as a descriptive summary and thematically analyzed using a case study approach. Data from the matrix and discussions with key informants were used to prepare case studies for each initiative. To understand the potential of each initiative, SWOT (Strength, Weaknesses, Opportunities and Threat) analysis [22] was done using the WHO building blocks of a health system as categories [23]. To improve methodological rigor, a final round of consultations (optional in the framework) was undertaken. Representatives from the identified initiatives were invited to review the findings. Disagreements were explored until consensus was reached (Table 2).

**Table 2:** Identified eHealth and mHealth initiatives in Bangladesh by managing entities (Data valid till March 2012)

**Status**	**Name**
**Public (4, 15.4%)**	
✓	DGHS DHIS-2
✓	DGHS OAMS
✓	DGHS MPHS
✓	DGHS Telemedicine
**Private (for profit) (14, 53.8%)**	
✓	Medinova Telemedicine
✓	eClinic24 (Chakaria Project) by TRCL
✓	AMCARE by TRCL
✓	Health services for the expats in Singapore by TRCL
X	Breast Cancer Finding via mobile by Amader Gram
X	JBFH Telemedicine
X	Friendship by mPower
✓	MHSBC by mPower
✓	Grameen Phone
~	Banglalink
~	Airtel
✓	Citycell
~	Robi
✓	TeleTalk
**Private (not for profit) (4, 15.4%)**	
~	mCare by JHSPH
~	mTIKKA by JHSPH
✓	MJiVita by JHSPH
X	SAJIDA Mobile Telemedicine
**NGO (4, 15.4%)**	
✓	CRP Telemedicine
✓	infoLADY by DNet
✓	MAMA by DNet
✓	BRAC m health

✓Ongoing. X Completed ~ Still in Development.

Accessing information was very challenging from the private sector actors who were, apart from a few exceptions, unwilling to share data. Information on public sector interventions was accessed through both formal and informal channels. NGOs were the most forthcoming participants in the study. Where data was not available, information was obtained from published sources, reports and web pages.

## Results

The year 1998 is a milestone for eHealth in Bangladesh as the first eHealth project was launched by *Swinfen Charitable,* a not-for-profit institute. It involved a collaboration between the Centre for the Rehabilitation of the Paralyzed (CRP) in Bangladesh and the Royal Navy Hospital Haslar, in UK. During the same year, the Ministry of Health and Family Welfare (MoHFW) initiated their first eHealth initiative [24]. Just a year later the Telemedicine Reference Center Limited (TRCL), a private company, initiated the use of mobile phones for healthcare delivery. In 2001, a professional coalition was established, the Bangladesh Telemedicine Association (BTA). This provided a platform for the ongoing and sporadic eHealth initiatives in the country. A similar platform called the Sustainable Development Network Program (SDNP) was formed in 2003, aimed at establishing better collaboration and understanding between providers [3]. Later in 2006, TRCL paired with GrameenPhone (GP) and initiated a mobile phone based call center for subscribers called Health Line:789 [25]. A number of NGOs, including BRAC, Sajida Foundation and DNet subsequently developed an interest in eHealth and mHealth. The main focus of their interest was on enhancing the efficiency of project implementation, for example by monitoring and evaluating interventions. Later many private entities became involved in telemedicine and/or patient record systems in their clinics and hospitals.

There are incidences of discontinued interventions as well. Our findings identified two: the joint telemedicine service operated by Bangladesh University of Engineering and Technology (BUET) and Comfort Nursing Home, established in 2003; and the Bangladesh DNS Diagnosis Centre (2004) [3]. The principle reasons given for discontinuation included financial problems, poor marketing and lack of demand.

In total, the study identified 26 initiatives (either pilot or full scale programs) with direct or indirect associations with eHealth and/or mHealth. The earliest telemedicine projects surfaced in the late 1990s [26]. Table 2 lists these initiatives: four public; eighteen private; and four NGO. Seventeen were ongoing projects at the time of this review, mostly in an early phase, though some had been operating for over 5 years and other close to cessation. Nine ongoing projects were scaled up versions of successful pilots. Following discussions, these initiatives were grouped as per WHO health system building blocks.

### Health service delivery

**Figure 2:**
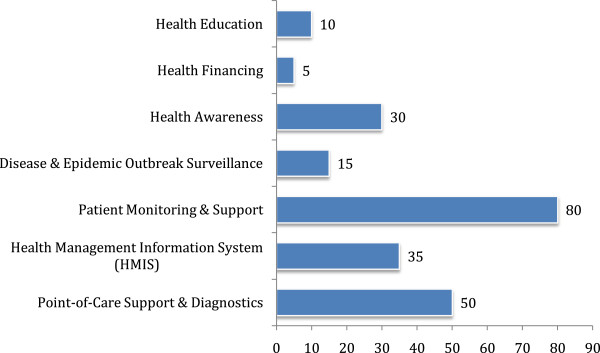
Services offered by eHealth initiatives.

All the listed initiatives had a mandate to deliver health services and/or manage health information (described later). Figure 2 shows the various health services offered. The primary form of service delivery was tele-consultation, which included remote diagnosis through video conferencing and imaging, and advice on disease management, prescriptions and/or referral. Two types of referral were generally practiced; generic and specific. In most cases patients were advised to attend any health facility that offered the required care. Specific referral was more common in private telemedicine services where patients were referred to their empanelled health facilities depending on the location of the caller. TRCL’s initiative provides an excellent example of this approach. Among the NGOs, DNet had similar links with partners for service provision, mostly to other NGOs who offered various levels of services. Others including BRAC, SAJIDA Foundation and CRP referred clients to their own facilities for higher care.

### Health information

As indicated above, the other main use of eHealth is health management, typically linked to an information system. Computerized Health Management Information Systems (HMIS) typically have remote and central data entry, compilation and output interfaces. In recent times most projects have developed (or are in the process of developing) mobile and/or cloud based web applications appropriate for their end-users and tailored to their service provision needs. These now play an important role in the management of public health in Bangladesh. All public health facilities are networked and linked to the Directorate General of Health Services (DGHS). This helps in periodic reporting of health indicators across the country. HMIS for both health services and administration provides the basis for the collection, compilation and reporting of a range of health statistics. Eight of the listed initiatives have specific HMIS, including the four public sector ones.

In addition, most of the initiatives have some form of information management system to track their own activities. However, there was no evidence which documents effective sharing of information and experiences. Currently there is no forum/body to serve as a repository for this sharing of information, except for a government initiative (implemented by the DGHS) that receives data from various health facilities across the country. The government has also initiated a database of all public health professionals.

### Human resource for eHealth

There is no effective policy relating to Health Human Resources (HHR) in Bangladesh. In the case of eHealth, the roles and types of human resource required are also not well defined. Academic courses are crucial for developing skills and competencies but currently only one private medical university post-graduate institution, the Bangladesh Institute of Health Science (BIHS), offers a program on health informatics where eHealth is a major topic. The government has however conducted several capacity building workshops of various lengths for health professionals over 2010–2012 under the Bangladesh Health, Nutrition and Population Sector Program (HNPSP), UNICEF and WHO [27]. Beyond this, all relevant non-government and private organizations doing eHealth projects, train their staff in line with available practice, ranging from basic medical training to hands on sessions on the use of technical materials and technological platforms. These courses do not have a specific long-term vision or goal relating to eHealth and most are informal, i.e. without professional certification or accreditation.

The current pool of human resources (HR) for eHealth and mHealth is therefore very limited given the number of interventions. The findings of this study revealed that 18 private and 4 NGO initiatives were employing 2 to 75 staff with a wide range of academic and professional backgrounds, e.g. doctors and medical assistants, health workers and IT and software personnel. The government has 721 sanctioned posts across the country with a vacancy rate of 26% [27].

### Technology

Currently, telecommunications is the predominant technological platform for eHealth and mHealth based service delivery in the country, mainly established around consultation via call centers and SMS. A substantial proportion of the population (in October 2012 it was 98 million [28] approximately two-thirds of the total) has physical access to such tele-consultation. The commercial and more clinical initiatives are mostly using a combination of internet and mobile technology. Areas covered within these initiatives include raising health awareness, providing electronic prescriptions, creating vaccine registries, using videos & still images for diagnosis etc. Our findings suggest that video is the least used mode although it has the potential for sharing synchronous or real time visual information and has the ability to be used for remote diagnosis based on visual interpretation.

### Financing

According to the global e-Health survey conducted by WHO [29], financial management in eHealth and mHealth is largely met by donors, either directly or through management agencies. Thus they are collectively meeting expenditures for activities on health ICT and the associated capacity building. Government eHealth and mHealth projects are mainly financed from the national health budget. In addition, ICT also received a collateral allocation (approximately USD 38 million) from the Ministry of Information Communication Technology (national budget, 2012–2013) [30,31]. However, the exact share allocated for eHealth and mHealth is hard to identify since it remains as an embedded component. In recent days, eHealth and mHealth projects are being allocated to public-private partnership. Donor groups include Swinfen Charitable Trust, Rockefeller Foundation and Johns Hopkins Bloomberg School of Public Health.

As indicated, eHealth and mHealth is largely dominated by private for profit entities and thus not dependent on the donors. However, only four initiatives reported sustainability plans during our survey. Further discussions revealed that since sustainability was linked to financial profile (i.e. profit, expenditure, tax), commercial enterprises were unwilling to share such information publicly or to the researchers.

### Leadership and governance

To understand governance mechanisms in eHealth and mHealth, existing strategy and policy documents were analyzed. Currently there is no standard or widely accepted operational framework for eHealth or mHealth in the country. As a result, it is very difficult to assess the comparative health gains, e.g. percentage of people with access to eHealth/mHealth services or attributable change in national health indicators, etc. Furthermore, there is no working collaboration between the ministry of health and the ministry of ICT, resulting in a vertical approach to the implementation of projects. Further analysis identified an absence of legal and ethical frameworks for eHealth, for example relating to personal health data, internet safety or equity. Creating practical indicators to measure and assure the quality of eHealth and mHealth services is of the utmost importance. This was reflected in findings suggesting that a minority of the listed projects reported having monitoring and evaluation frameworks and less than one quarter had undertaken an evaluation for effectiveness. Such evaluation or monitoring documents that did exist were not accessible.

## Discussion

At a juncture when Bangladesh has adopted the United Nations resolution for Universal Health Coverage, much is being said about the potential for eHealth and mHealth. But is there any evidence that this can have a positive impact? The findings of this study suggest that initiatives have proliferated predominantly in the private sector, with most being linked to mobile phones. It is almost impossible to gauge their effectiveness in terms of health outcomes. In addition differences in perceptions of eHealth and mHealth across the country (and globally) have contributed to a wide variation in system designs and implementations, making comparative evaluation extremely difficult. It is encouraging that the government has made serious attempts to integrate public sector information systems. They have stepped in early and gained considerable ground in this regard. Their early adoption of the technology also promotes acceptance of their active role in providing guidance through the introduction of a simple and strategic framework for eHealth. It should also enable us to compare the effectiveness of different eHealth and mHealth initiatives in achieving health system goals.

The growing investment in ICT and the telecommunications industry has seen a substantial diminution of ‘technophobia’ among the general public, and has improved access, especially for the poor [31]. With rising acceptability, eHealth and mHealth initiatives are steadily emerging in both developing and highly industrialized countries, though with variable and sometimes counter-intuitive outcomes. For example, while there is evidence that an SMS based sentinel system has substantially improved disease surveillance in Madagascar [32], the highly sophisticated UK National Health System recently had to abandon a major national IT program to centralize patient records in midcourse [33,34]. In Bangladesh, WHO reported that the MoHFW reached 98% of its target population through SMS on health education but did not provide messages in Bangla, which is the first language of the majority of the population [14]. Despite the glorified prospects for ICT in health systems, one has to be careful about the challenges in terms of “evidence, sustainability, human resources, funding, interoperability, ICT infrastructure, legal and ethical constrains” [35].

Thus far it is not clear, from the sporadic and disjointed initiatives, how eHealth has been assimilated to strengthen particular health system goals for Bangladesh. The experience in this paper suggests that it is time for Bangladesh to start making moves toward a national eHealth/mHealth policy and a national telemedicine policy addressing all the building blocks of the health system. The Rockefeller foundation emphasizes the need for a national eHealth policy and provides a review on nine fundamental points to be considered [36]. Such policies are also important to align eHealth and mHealth with the goal of Universal Health Coverage [35]. The WHO/ITU toolkit [37] provides a map of possible eHealth solutions to be integrated in a health system and would appear to be a reasonable starting point, with the government prioritizing those that are practicable at this stage.

There is also considerable uncertainty as to the most appropriate design of eHealth and/or mHealth initiatives with regard to the mode and content of health service delivery. The single most popular service appears to be tele-consultation, which is probably an underutilization of the potential of eHealth and mHealth. In other parts of the world there are examples of virtual doctors and self-conducted computer guided checkup & diagnostic systems [38]. Initiatives for referral also hold much potential especially in overcoming geographic barriers. These warrant more research. If properly installed, they can be used for real time, end-to-end tracking of referred cases which is a huge challenge in the current system [39]. Real time clinical services for remote and hard to reach areas also merit further research especially in regard to how such eHealth/mHealth platforms can complement the existing healthcare initiatives. ICT based notification systems for public health emergencies and diseases are yet to be explored to their full potential.

The quality of all eHealth and mHealth services also requires much attention. Although quality was not directly measured, it can be extrapolated from our findings that most of the eHealth initiatives are deficient in one or more of the following service quality parameters [40]: knowledge and competence of provider; capacity of access and monitoring devices; operational compatibility and information interoperability. Human resource plays a central role in determining the potential effectiveness of eHealth and mHealth based interventions. There needs to be considerable investment in creating capacity in this area, both to design and manage initiatives. In the current context, the establishment of professional courses could play a key role in better positioning eHealth and mHealth as a tool for health service and integrating it with the existing health system. A generic document that described the required competencies in this area would be a valuable first step.

One of the challenges of this study was to obtain information from the private sector. In theory the use of ICT can improve transparency and accountability if linked to reliable and timely information sharing. If the private sector does not share information on its projects, then it actually undermines the development of ICT services. With appropriate policies in place, private sector actors could be made more accountable.

It is of the utmost importance to conduct further research on eHealth and mHealth and identify their appropriate application in Bangladesh. In addition to describing the possible designs and modes of service delivery, it will also be imperative to determine the acceptability of eHealth and mHealth initiatives to the general population. Data security will be a central issue that requires considerable attention. The Constitution of Bangladesh recognizes the right to privacy. However, the existing legal framework does not include a general data protection act. If consumers could be assured that sensitive health data would be held securely, their attitude towards eHealth and mHealth initiatives would be considerably more relaxed. Thus an important first step would be to enact and implement appropriate regulation. Adopting the national eHealth strategy toolkit developed by the WHO International Telecommunications Union [37] would address issues related to privacy, transparency and accountability.

## Conclusion

Our present analysis brings us to the conclusion that it is too early to assess the effectiveness of the application of eHealth and mHealth initiatives in Bangladesh. The growing interest of the private sector in this area suggests a strong inclination for significant expansion. It also suggests the potential financial viability of eHealth and mHealth initiatives. However, it is yet not clear how eHealth and mHealth will be integrated into the existing health system given the dearth of reliable evidence. Based on what is available, the most urgent area for research will be how to devise a framework to ensure effectiveness, accountability and equity in delivering services through this platform. Considering the experience gained over the last decade, Bangladesh is perfectly positioned to develop such a framework. Involvement of the public sector will help in generating evidence on the most effective means of integrating eHealth and mHealth into health systems. With the current political mandate of *Digital Bangladesh,* a logical step forward for the government would be to become the steward of eHealth and mHealth and support the link between technology and health in this country.

### Limitation

This scoping study puts forward some evidence on current eHealth and mHealth initiatives in Bangladesh and their potentials as a health system tool. We recommend future in-depth studies on these initiatives which would guide the development of conceptual and legal frameworks for health system integration which was not possible in the current design.

## Competing interests

The authors declare that they have no competing or financial interests.

## Authors’ contributions

TA and HL, both were involved in every aspect of the project and the manuscript; starting from conceptualization of the project and its implementation, from drafting of the high level outline of the manuscript to finalization and submission. ASK and RI were chiefly involved in implementation of the project and then the manuscript. AB is a very senior health system scientist and has provided the overall guidance and mentorship to the project. MI is the principal investigator of the project and has been a leader in all the aspects of the entire endeavor. All authors read and approved the final manuscript.

## Pre-publication history

The pre-publication history for this paper can be accessed here:

http://www.biomedcentral.com/1472-6963/14/260/prepub
